# Transgenic mice overexpressing miR-137 in the brain show schizophrenia-associated behavioral deficits and transcriptome profiles

**DOI:** 10.1371/journal.pone.0220389

**Published:** 2019-07-30

**Authors:** Yuuichi Arakawa, Kazumasa Yokoyama, Shinya Tasaki, Junichi Kato, Kosuke Nakashima, Michiyasu Takeyama, Atsushi Nakatani, Motohisa Suzuki

**Affiliations:** 1 Neuroscience Drug Discovery Unit, Research, Takeda Pharmaceutical Company Limited, Fujisawa, Kanagawa, Japan; 2 Integrated Technology Research Laboratories, Pharmaceutical Research Division, Takeda Pharmaceutical Company Limited, Fujisawa, Kanagawa, Japan; University of Queensland, AUSTRALIA

## Abstract

Schizophrenia is a psychiatric disorder characterized by positive and negative symptoms and cognitive deficits. The exact cause of schizophrenia is still unknown, but substantial evidence indicates that it has a genetic component. Genome wide association studies demonstrate variants within miR-137 host gene are a risk factor for schizophrenia. However, the direct relationship between the pathophysiology of schizophrenia and the dosage of miR-137 remains unclear. Therefore, in this study, we generated transgenic mice overexpressing miR-137 (miR-137 Tg mice) with the neuron-specific Thy-1 promoter and examined schizophrenia-related phenotypes in these mice. Overexpression of miR-137 was observed in various brain regions of the miR-137 Tg mice, with down-regulation of putative miR-137 targets. MiR-137 Tg mice showed sensory gating deficits in a prepulse inhibition test, social deficits in a sociability and social novelty test, and cognitive deficits in a novel object recognition test. Interestingly, the predicted-altered pathways of the medial prefrontal cortex of miR-137 Tg mice were partially overlapped with those of the dorsolateral prefrontal cortex in postmortem brain of patients who died in equal to or less than 4 years after initial diagnosis of schizophrenia in published data. These results suggest that overexpression of miR-137 in the whole brain induces the several phenotypes that are relevant to aspects of psychiatric disorders, such as schizophrenia. Based on these findings, miR-137 Tg mice may have the potential to become a useful tool in researching the pathophysiology of psychiatric disorders.

## Introduction

Schizophrenia is a psychiatric disorder characterized by positive and negative symptoms and cognitive deficits [1], and is thought to result from a complex interplay of genetics and environmental factors [2, 3]. Functional magnetic resonance imaging and electroencephalogram studies of patients with schizophrenia show abnormal neural activities in various brain regions, such as the prefrontal cortex (PFC), hippocampus, thalamus, striatum, and cerebellum [4–6]. To date, the results of genome-wide association study (GWAS) analyses indicate an involvement of multiple and heterogeneous genetic factors in schizophrenia [7, 8].

It is demonstrated that the single nucleotide polymorphism (SNP) within the miR-137 host gene (MIR137) has the strongest genetic association with schizophrenia [9]. In the largest GWAS for schizophrenia to date, SNPs within MIR137 are the second most significant association after the major histocompatibility complex region [10]. MiR-137 is a brain-enriched miRNA that is highly expressed in whole brain regions except for the cerebellum [11]. MiR-137 can modulate the expression of multiple genes and the activity of pathways [12] which play important roles in regulating the development of the embryonic neural stem cell, neuronal proliferation and differentiation, and synaptic maturation [13]. Multiple GWAS analyses in schizophrenia demonstrate SNPs (rs1625579, rs2660304 and rs1198588) within MIR137 are associated with schizophrenia [9, 10, 14, 15]. Moreover, rs1625579 within MIR137 is genetically associated with several phenotypes of schizophrenia, including cognitive deficits and negative symptoms [16], as well as earlier age at onset [17]. Most of expression quantitative trait locus studies for miR-137 indicate that major alleles, higher risk of schizophrenia, decrease miR-137 expression [11, 14, 15], including a study that minor alleles increase miR-137 expression [18]. Meanwhile, the expression of miR-137 in postmortem brain samples does not change in the patients with schizophrenia compared to healthy control [11, 19]. On the other hands, recent studies have demonstrated that the expression of miR-137 in peripheral blood was increased in the first episode patients with schizophrenia compared to those of healthy controls [20, 21]. Thus, the direct relationship between the pathophysiology of schizophrenia and the dosage of miR-137 remains to be unclear.

Lentivirus-mediated overexpression of miR-137 in the mouse hippocampal dentate gyrus down-regulates the expression level of presynaptic genes and impairs the induction of mossy fiber long-term potentiation, which results in deficits in hippocampus-dependent learning and memory [18]. However, the effects of miR-137 overexpression in the whole brain still remain unknown. Therefore, we generated miR-137 transgenic mice (miR-137 Tg mice) in which miR-137 is expressed in the whole brain regions with the neuron-specific Thy-1 promoter [22], and examined the effects of overexpressed miR-137 on psychiatry-like phenotypes, especially schizophrenia related-phenotypes, such as positive and negative symptom-like behavior and cognitive deficits. Moreover, we performed a transcriptional analysis comparing with public data with postmortem brain samples from patients with schizophrenia.

## Materials and methods

### Animals

Wild-type (WT) mice and miR-137 Tg mice were housed in groups of 2-4/cage in a light-controlled room (12-hour light/dark cycle, with lights on at 7 AM) and fed with free access to food and water. After an acclimation period of at least 1 week, male mice at between 3 to 10 months old were used. The care and use of the animals and the experimental protocols were approved by the Institutional Animal Care and Use Committee of Takeda Pharmaceutical Company Limited (protocol number: 00010713). Animal research facilities in Takeda Pharmaceutical Company Limited (Kanagawa, Japan) are accredited by the Association for Assessment and Accreditation of Laboratory Animal Care (AAALAC). All efforts were made to minimize suffering.

### Generation of miR-137 Tg mice

The 946 bp genome fragment encompassing murine mature miR-137 (NCBI Gene ID: 387155) was cloned by PCR and ligated with Thy-1 promoter. The pThy1.2 expression cassette was gifted from Dr. Pico Caroni at the Friedrich Miescher Institute, Basel, Switzerland [23]. The resulting Thy-1-miR-137 plasmid was linearized by digestion with restriction enzymes (EcoRI and PvuI) and was then microinjected into fertilized eggs of the C57BL/6J mice (CLEA Japan, Tokyo, Japan). F1 pups were obtained from F0 transgenic founder mice by *in vitro* fertilization and were analyzed for the expression of miR-137 in the brain as described below.

### List of predicted miR-137 targets

MiRNAs are shown to bind to specific seeding sites in the 3' UTR [24]. MiRWalk is the comprehensive miRNA-target prediction database summarizing the results of several databases with a different algorithm [25]. We used miRWalk ver. 2.0 and counted the predicted number as hsa- or mmu-miR-137 target genes in 12 databases (miRWalk, Microt4, miRanda-rel2010, mirbridge, miRDB4.0, miRMap, miRNAMap, Pictar2, PITA, RNA22v2, RNAhybrid2.1 and Targetscan6.2). Genes predicted as miR-137 targets in more than 5 databases were used as the miR-137 target genes in this study.

### RNA extraction and quantitative real-time PCR

The mice at 3 and 10 months old were sacrificed by decapitation, and brain tissue from the medial PFC (mPFC), striatum, thalamus, hippocampus, and cerebellum was quickly dissected, frozen on dry ice, and stored at −80°C. Total RNA was extracted using the miRNeasy mini kit (Qiagen, Hilden, Germany) following the manufacturer's instruction. Reverse transcription was performed using the TaqMan MicroRNA Reverse Transcription Kit (Thermo Fischer Scientific, Waltham, MA) and the TaqMan miRNA assay for mature miR-137 and snoRNA202 (Thermo Fischer Scientific). The TaqMan miRNA assay was performed using TaqMan PreAmp Master Mix (Thermo Fischer Scientific). The cDNA samples were subjected to real-time PCR using the ViiA-7 instrument (Thermo Fischer Scientific), and the threshold cycle (Ct) of miR-137 was normalized by that of snoRNA202.

### Global transcriptional expression

Total RNAs of the mice at 3 months old were used for this analysis and were quantified using Qubit 3.0 Fluorimeter (Thermo Fisher Scientific). Sequencing libraries were prepared using the Ion Total RNA-Seq Kit v2 (Thermo Fisher Scientific) from total RNA, according to the manufacturer’s instructions. Quantification of the amplified cDNA was performed on an Agilent 2100 Bioanalyzer with the Agilent High Sensitivity DNA Kit (Agilent Technologies, Santa Clara, CA). Libraries were pooled equally with six samples. Emulsion PCR, enrichment, and loading were performed on an Ion Chef Instrument (Thermo Fisher Scientific) using the Ion PI Hi-Q Chef Kit (Thermo Fisher Scientific) and the Ion PI Chip Kit v3 (Thermo Fisher Scientific). The samples were then sequenced on an Ion Proton System (Thermo Fisher Scientific) using the Ion PI Hi-Q Sequencing 200 Kit (Thermo Fisher Scientific).

### Comparison of transcriptional changes

Throughout the analysis, genes were selected if they showed a count >0 in at least 20% samples. The read count was transformed to the fragment per kilo million values. The transcriptional changes between WT mice and miR-137 Tg mice were analyzed by Aspin-Welch test. We set the criterion for statistical significance at RPKM (reads per kilobase of exon per million mapped reads) >0.5, *p*-value <0.05, and absolute fold change >1.2.

The Correlation Engine software (Illumina, San Diego, CA) enables ontology-based meta-analysis of global collections of the public available gene expression datasets by using the pairwise rank-based enrichment analysis [26]. The transcriptome data obtained from miR-137 Tg mice were imported into Correlation Engine (Illumina) and were compared with the bioset of mmu-miR-137 putative target genes selected by miRWalk ver.2.0. Pairwise rank-based enrichment analysis was performed by the Correlation Engine software, and enrichment *p*-value was calculated (Illumina).

To find biosets whose changing expression is highly overlapped with miR-137 Tg mice, we used the Correlation Engine database of patients with schizophrenia (Illumina). Public transcriptome data was used under the condition of *p*-value <0.05 and absolute fold change >1.2. We selected a similar bioset showing transcriptional changes of patients with schizophrenia compared with healthy control subjects. Next, we compared the selected biosets with the bioset of hsa-miR-137 target genes selected by miRWalk ver. 2.0. Pairwise rank-based enrichment analysis was performed by the Correlation Engine software, and enrichment *p*-value was calculated (Illumina).

Finally, biosets showing transcriptional changes in miR-137 Tg mice and patients with schizophrenia were imported into Ingenuity Pathway Analysis (IPA) (ver. 47547484) (Qiagen). Core analysis in the IPA software predicted canonical pathways that are changing based on gene expression. Pathway activation z-score was calculated and predicts whether the pathway was up-regulated or down-regulated. The positive number indicates that the pathway was up-regulated, and the negative number indicates that the pathway was down-regulated. Pathway activation *z*-scores showing greater than 2 or smaller than −2 were judged as significant. Comparisons of the canonical pathways between miR-137 Tg mice and patients with schizophrenia were performed by comparison analysis in the IPA program (Qiagen).

### Prepulse inhibition (PPI) test

The PPI test was performed using 4-month-old mice. The experiments employed 8 SR-LAB acoustic startle chambers (San Diego Instruments, San Diego, CA). Each chamber consisted of a clear Plexiglas cylinder resting on a Plexiglas platform inside a ventilated enclosure housed in a sound-attenuated room. High-frequency speakers mounted above the cylinders produced all acoustic stimuli. Piezoelectric accelerometers mounted under the cylinders transduced movements of the animals. During the test sessions, individual mice were placed in the startle chambers and the background noise (70 dB) was initiated. After a 5-min acclimation period, each subject was presented with 54 trials having variable inter-trial intervals (7–23 s). The trials consisted of the following three types: (1) a pulse-only trial of 118 dB presented for 40 ms, during which the startle response was recorded; (2) two prepulse trial types consisting of 118 dB presented for 40 ms, which was preceded 100 ms earlier by a 20-ms prepulse of 76 or 82 dB. Startle response was recorded for 40 ms starting at the onset of the 118-dB pulse; and (3) a no-stimulus trial in which only background noise was presented. The percentage of PPI of the 76 or 82 dB prepulse was calculated using the following formula:

$$\text{\%PPI=}\frac{\begin{array}{l} \text{(average maximum starle of pulse only trials at 118 dB)}\\ -\\ \text{(average maximum starle of prepulse trials)} \end{array}}{\text{(average maximum startle of pulse only trails at 118 dB)}}\text{x100}.$$

### Sociability and social novelty test

The sociability and social novelty tests were performed using 6-month-old mice. The apparatus was a three-chamber grey acryl box (center chamber: 14.5 × 19 × 21.5 cm, outer chamber: 19.3 × 19 × 21.5 cm), and dividing walls were made from clear acryl plates with 8-cm-wide gates. In the two outer chambers, transparent cylinders with small holes (8.2 cm ϕ × 20.5 cm, hole: 1.3 cm ϕ) were placed to avoid direct physical interaction between mice. The target animals were age-matched WT mice that had no previous contact with test animals. The mice were habituated to the test arena without cylinders for 5 min with their cagemates 2 days before the test, and then were individually habituated to the test arena with cylinders for 5 min 1 day before the test. On the test day, each test animal was placed in the middle chamber of test apparatus for 3 min with the gate closed by partition. The target 1 mouse was introduced in one of the cylinders while the other was kept empty. The partitions were then removed gently, allowing the test animal to explore freely all three chambers for 5 min (sociability test). The test mouse was placed in the center area again and the gate was closed for 30 s, during which time another mouse was placed into the previously empty cylinder (termed as the target 2 mouse). The partitions were removed and the test mouse was allowed to explore another 5 min (social novelty test). The test mouse was observed for the total sniffing duration (defined as having the nose in contact with the cylinder). The sniffing index was calculated as the index of sociability in the social approach test and social recognition in the social novelty test using the following equation:

$$\text{Sniffing index (sociability test)}=\frac{\text{Sniffing time in cylinder with target 1 (s)-Sniffing time in empty cylinder (s)}}{\text{Total sniffing time (s)}}$$

$$\text{Sniffing index (social novelty test)}=\frac{\text{Sniffing time in cylinder with target 2 (s)-Sniffing time in cylineder with target 1 (s)}}{\text{Total sniffing time (s)}}$$

### Novel object recognition (NOR) test

The NOR test was performed using 9-month-old mice. The test consisted of two parts: the habituation and test sessions (acquisition trial and retention trial). We used a gray 30 × 30 × 30 cm test box, a silver-aluminum square pole and a gray vinyl chloride circular cone as objects. In the habituation session on day 1, each mouse was habituated to the empty test box by placing it in the box and allowing it to explore for 5 min. In the test session for the acquisition trial on day 2, two identical objects were symmetrically placed in the corner of the test box. Each mouse was placed in another corner of the box with their head pointing toward the corner. Five minutes were allowed to explore each object, followed by a 24-hour retention interval in the home cage. In the test session for the retention trial on day 3, the mouse was placed back into the same box, where one of the familiar objects used during the acquisition trial was replaced with a novel object. The mouse was allowed to explore freely for 5 min. A preference ratio of the time exploring the novel object to the time exploring both objects was calculated as an index of cognitive function:

$$\text{Novelty discrimination index (NDI)}=\frac{\text{exploring time of novel object}}{\text{exploring time of both objects}}\;\times \;100\;\left(\text{\%}\right)\;.$$

### Statistics

Data were presented as mean + standard error of the mean (SEM). The statistical difference between the two groups was analyzed using the Student's *t*-test or Aspin-Welch test, and significance was set at *p* <0.05. All analyses were performed using the SAS system 8 (SAS Institute, Cary, NC).

## Results

### Generation of miR-137 Tg mice

**Fig 1:**
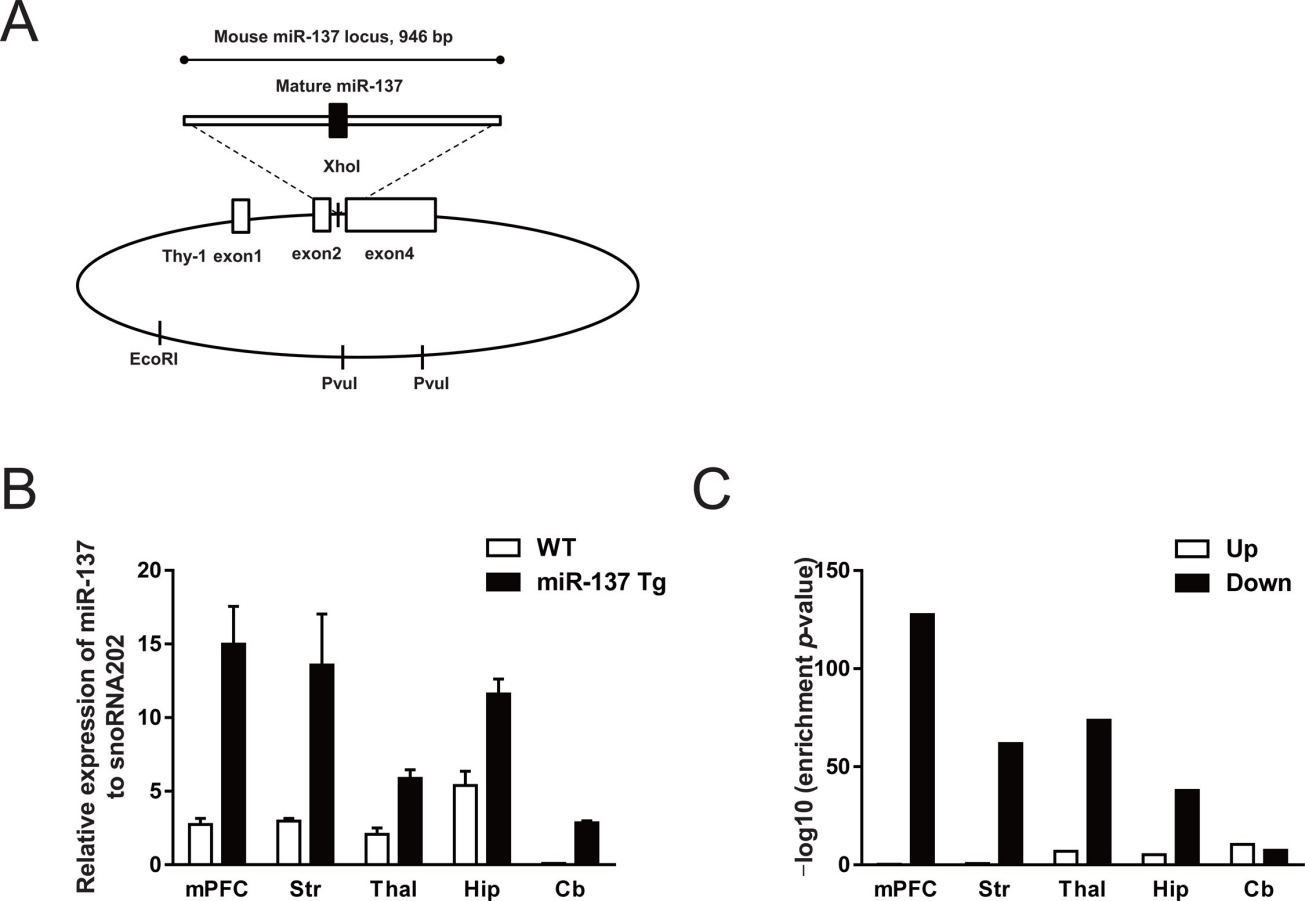
Generation of miR-137 Tg mice and altered gene expression of the putative targets for miR-137. (A) Transgene constructs. The genomic region encompassing mature miR-137 was ligated into the XhoI site of the plasmid containing the Thy-1 promoter. The Thy-1 promoter/miR-137 plasmid was linearized using restriction enzyme digestion and was microinjected into C57BL/6J fertilized eggs. (B) Expression level of miR-137 in several brain regions in WT and miR-137 Tg mice. Data are expressed as the mean + SEM (3-month-old male mice, n = 3). (C) Comparison of transcriptional changes of the miR-137 Tg mice at 3 months of age and mmu-miR-137-predicted target genes. Enrichment *p*-values were calculated in each brain region. mPFC, medial prefrontal cortex; Str, striatum; Thal, thalamus; Hip, hippocampus; Cb, cerebellum.

We generated transgenic mice overexpressing miR-137 using a neuron-specific Thy-1 promoter (Fig 1A) and measured the expression of miR-137 in various brain regions at 3 months old. In WT mice, the miR-137 was obviously expressed in the various regions, such as mPFC, striatum, thalamus, and hippocampus, but was only slightly detected in the cerebellum (Fig 1B). Compared with WT mice, miR-137 Tg mice showed an apparent overexpression of miR-137 in all examined brain regions (Fig 1B). Moreover, the expression levels of miR-137 in miR-137 Tg mice at 10 months old were increased at a degree similar to that seen in miR-137 Tg mice at 3 months old as compared with the WT mice (S1 Fig). These results suggest that miR-137 Tg mice overexpress miR-137 in the whole brain including the cerebellum from 3 to 10 months of age.

### Down-regulation of miR-137 putative target genes in miR-137 Tg mouse brains

To assess the effects of overexpressed miR-137 on the gene expressions of miR-137 putative targets, we examined the transcriptome analysis in several brain regions. The number of genes whose expression levels were changed in the brain tissue of miR-137 Tg mice comparing with that of WT mice was as follows: mPFC (up: 561, down: 1287), striatum (up: 748, down: 294), thalamus (up: 1087, down: 563), hippocampus (up: 471, down: 402), and cerebellum (up: 371, down: 617) (S1 Table). The miRWalk ver. 2.0 dataset was used to select 2356 genes as the putative target genes, which were predicted in more than 5 databases as the miR-137 target (S2 Table), and we used them in analysis of miR-137 Tg mice. The number of putative mmu-miR-137 target genes showing the altered expression level was as follows: mPFC (up: 22, down: 365), striatum (up: 37, down: 102), thalamus (up: 97, down: 146), hippocampus (up: 43, down: 90), and cerebellum (up: 46, down: 59) (S3 Table). There was a strong overlap between down-regulated genes and putative miR-137 target genes in pairwise rank-based enrichment analysis (enrichment *p*-value; 2.8E-128, 1.7E-62, 2.1E-74, 1.1E-38, and 3.5E-8 for mPFC, striatum, thalamus, hippocampus, and cerebellum, respectively; Fig 1C). In contrast, compared to those of down-regulated genes, there was a weaker overlap between up-regulated genes and putative miR-137 target genes, except for the cerebellum (enrichment *p*-value; 0.50, 0.18, 1.2E-7, 5.9E-6 and 3.5E-11 for mPFC, striatum, thalamus, hippocampus, and cerebellum, respectively; Fig 1C). Brain transcriptome analysis showed that the overexpressed miR-137 significantly reduced the expression level of the putative target genes.

### Behavioral alterations in miR-137 Tg mice

It is of interest that overexpression of miR-137 in the brain leads to behavioral changes associated with the core symptoms of patients with schizophrenia. We tested the performance of miR-137 Tg mice in the PPI test, sociability and social novelty test, and NOR test as benchmarks of positive symptoms, negative symptoms, and cognitive deficits, respectively [27–29].

**Fig 2:**
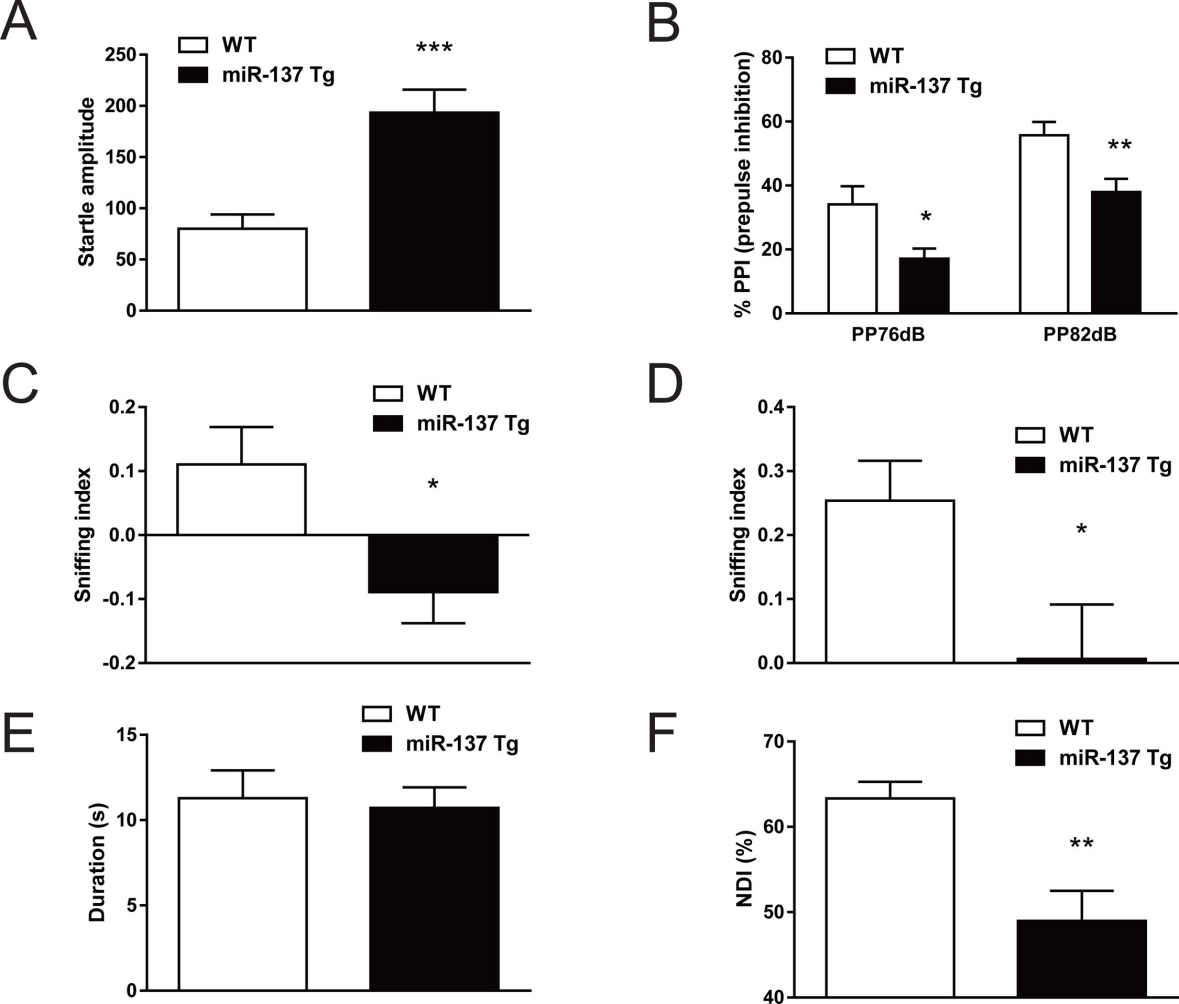
Behavioral analysis related to core symptoms of schizophrenia. (A, B) PPI test, (C) sociability test, (D) social novelty test, (E) NOR test (acquisition trial) and (F) NOR test (retention trial) in WT and miR-137 Tg mice. Data are expressed as the mean + SEM. (A, B) 4-month-old male mice, n = 12, ^*^p <0.05, ^***^ *p* <0.001 vs. WT (Aspin-Welch test), ^**^p <0.01 vs. WT (Student's *t*-test); (C, D) 6-month-old male mice, n = 11, ^*^p <0.05 vs. WT (Student's *t*-test); (E, F) 9-month-old male mice, n = 8, ^**^p <0.01 vs. WT (Aspin-Welch test).

In the PPI test, the miR-137 Tg mice exhibited a significant increase of startle amplitude (*p* <0.001; Fig 2A), whereas the miR-137 Tg mice exhibited a significant decrease of PPI (prepulse 76 dB; *p* <0.05, prepulse 82 dB; *p* <0.01; Fig 2B). And the miR-137 Tg mice exhibited a significant decrease of the sniffing index in both the sociability and the social novelty test (*p* <0.05), which are indices of sociability and social recognition, respectively (Fig 2C and 2D). In the NOR test, under the condition that total duration of object sniffing time in the acquisition trial did not change between WT mice and miR-137 Tg mice (Fig 2E), the miR-137 Tg mice exhibited a significant decrease of NDI (*p* <0.01), an index of cognitive function, compared with WT mice (Fig 2F).

### Overlapping transcriptional changes from the mPFC of miR-137 Tg mice with those of the dorsolateral PFC (DLPFC) in post-mortem brains of patients with schizophrenia

Functional abnormalities in the mPFC are implicated in phenotypes of schizophrenia [30]. The mouse mPFC shows to have anatomic and functional homology to the human DLPFC [31]. We compared transcriptomes from the mPFC of miR-137 Tg mice with data set related to schizophrenia in public by using the Correlation Engine database, resulting that most similar was those of the postmortem DLPFC from patients who died in equal to or less than 4 years after initial diagnosis of schizophrenia (defined as short duration of illness [short DOI]) in the report of Narayan et al [32]. That is, only direction showing down-regulated genes both in miR-137 Tg mice and the patients with schizophrenia with short DOI was strongly overlapped (enrichment *p*-value; 2.2E-25).

**Fig 3:**
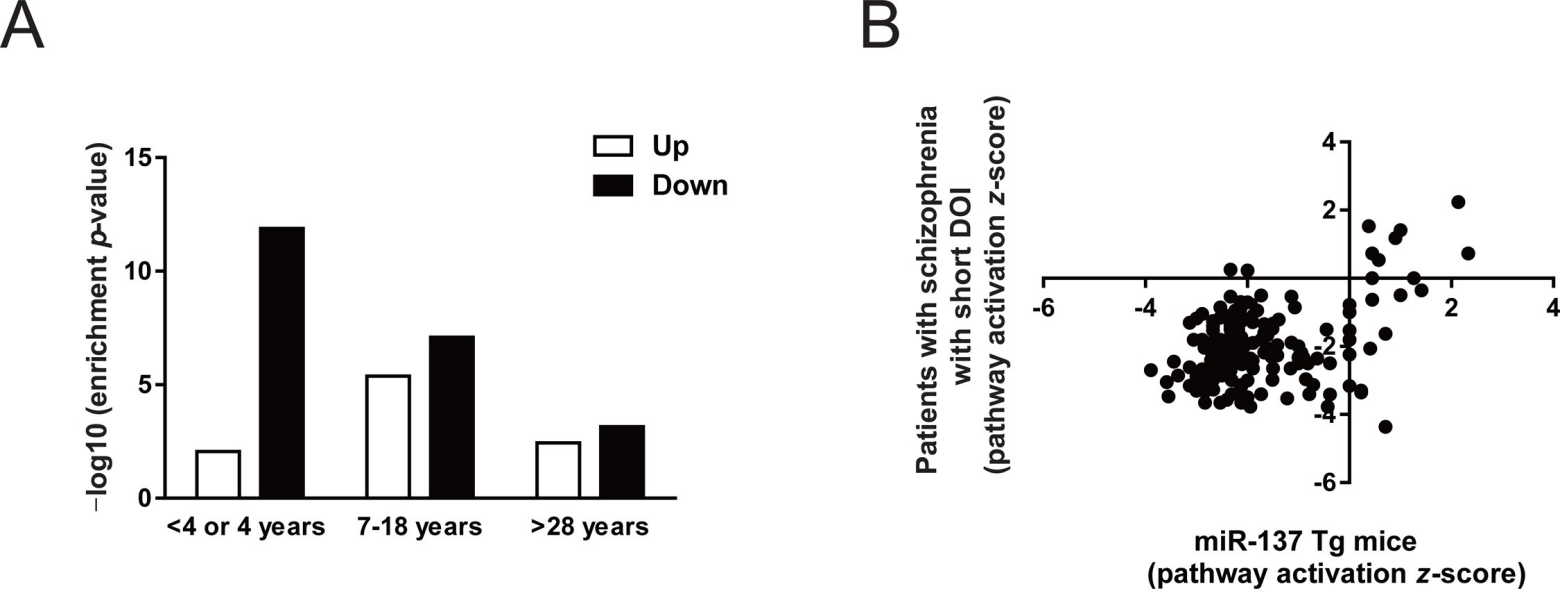
Comparison of transcriptional changes in patients with schizophrenia and miR-137 Tg mice. (A) Enrichment *p*-value calculated by comparing increased or decreased gene expression of miR-137-predicted targets in the mPFC of miR-137 Tg mice and in the DLPFC of postmortem brains in patients with schizophrenia who had different durations of illness. (B) Comparison of pathway activation *z*-score with patients who died in equal to or less than 4 years after initial diagnosis of schizophrenia and miR-137 Tg mice. The positive number indicates that the pathway was up-regulated, and the negative number indicates that the pathway was down-regulated.

Since the Correlation Engine also includes biosets of patients with schizophrenia with different DOIs in the report of Narayan et al [32], ranging 7–18 years from initial diagnosis to death (defined as intermediate DOI) and >28 years from initial diagnosis to death (defined as long DOI), we also compared the dataset from miR-137 Tg mice with the other two biosets. The number of genes whose expression levels were changed in the postmortem brain of the patients with schizophrenia comparing with that of healthy control was as follows: >28 years (up: 108, down: 137), 7–18 years (up: 685, down: 1059), ≤4 years (up: 781, down: 1644) (S4 Table). The miRWalk ver. 2.0 dataset was used to select 2714 genes as the putative target genes, which were predicted in more than 5 databases as hsa-miR-137 target (S5 Table), and we used them in analysis of the patient with schizophrenia. We observed an inverse overlap between the number of putative hsa-miR-137 target genes and DOIs (number of miR-137 target genes: >28 years (up: 19, down: 21), 7–18 years (up: 97, down: 204), ≤4 years (up: 73, down: 343) (S6 Table). Transcriptional changes of short DOI samples showed the strongest overlap between down-regulated genes and putative miR-137 target genes (enrichment *p*-value; 7.0E-4, 8.0E-8, and 1.3E-12 for >28 years, 7–18 years, and ≤4 years, respectively; Fig 3A). In contrast, transcriptional changes of short DOI samples showed the weakest overlap between up-regulated genes and putative miR-137 target genes (enrichment *p*-value; 3.6E-3, 4.1E-6, and 8.7E-3 for >28 years, 7–18 years, and ≤4 years, respectively; Fig 3A).

Finally, we performed a comparison of predicted-altered pathways that were based on transcriptional changes of short DOI samples and the mPFC of miR-137 Tg mice by using IPA software. Common canonical pathways in patients with schizophrenia with short DOI and miR-137 Tg mice were summarized (S7 Table). Most of the pathway activation *z*-score in common pathway showed the negative number, suggesting that these pathways were down-regulated (Fig 3B). Among them, pathway activation *z*-score of several common pathways showed smaller than −2, suggesting that alteration of these pathways were significant. Especially, the list of top 20 common pathways between the patients with schizophrenia and miR-137 Tg mice was summarized (Table 1). Synaptogenesis signaling pathway, an inflammatory-related signaling (CXCR4 signaling, P2Y purigenic receptor signaling pathway and IL-8 signaling) and the inter-cellular-related signaling (Gαq signaling and CREB signaling in neurons) were highly ranked.

**Table 1:** The list of top 20 common pathways between the patients with schizophrenia with short DOI and miR-137 Tg mice.

	Pathway activation *z*-score	
	miR-137 Tg mice	Patients with schizophrenia with short DOI
Synaptogenesis Signaling Pathway	-3.550	-3.474
Cardiac Hypertrophy Signaling	-3.578	-3.042
Cardiac Hypertrophy Signaling (Enhanced)	-3.889	-2.692
CXCR4 Signaling	-2.828	-3.657
AMPK Signaling	-3.000	-3.307
Gαq Signaling	-3.130	-3.157
P2Y Purigenic Receptor Signaling Pathway	-3.357	-2.858
Aldosterone Signaling in Epithelial Cells	-3.051	-3.153
Ephrin Receptor Signaling	-2.530	-3.656
Integrin Signaling	-2.837	-3.333
CREB Signaling in Neurons	-2.982	-3.128
IL-8 Signaling	-2.828	-3.182
Signaling by Rho Family GTPases	-2.400	-3.569
Role of NFAT in Cardiac Hypertrophy	-3.000	-2.967
Neuropathic Pain Signaling In Dorsal Horn Neurons	-2.668	-3.273
NF-κF Signaling	-3.441	-2.449
Apelin Endothelial Signaling Pathway	-3.000	-2.887
GNRH Signaling	-2.121	-3.651
Adrenomedullin signaling pathway	-3.130	-2.611
Rac Signaling	-1.941	-3.772

## Discussion

Here, we present the first characterization of miR-137 Tg mice at the behavioral and molecular levels. Overexpression of miR-137 was observed in various brain regions of the miR-137 Tg mice, with down-regulation of putative miR-137 targets. Behavioral experiments showed that the miR-137 Tg mice had psychiatry-like phenotypes, especially schizophrenia related-behavioral deficits in sensory gating, sociability, social recognition, and cognition. Interestingly, the predicted-altered pathways in the mPFC obtained from the miR-137 Tg mice were partially overlapped with those seen in the postmortem DLPFC of patients who died in equal to or less than 4 years after initial diagnosis of schizophrenia [32].

Previous study reports the overexpression of miR-137 in hippocampal dentate gyrus results in deficits in hippocampal-dependent learning and memory [18]. In this study, we confirmed that the miR-137 Tg mice display deficits in sensory gating, sociability and cognition, which are benchmarks of positive, negative symptoms and cognitive deficits, respectively (Fig 2A–2D and 2F) [27–29]. Sensory gating in the PPI test can be examined both in preclinical and clinical studies, and PPI of the startle response is associated with the mPFC, striatum, and thalamus [33]. Social recognition in human is associated with the cortex and amygdala [34], and the sociability and social recognition in rodents are involved in the mPFC [35]. Furthermore, the NOR test in rodents is considered to reflect visual learning and memory in humans [36], and multiple brain regions, such as hippocampus, insular cortex, perirhinal cortex, and mPFC are associated with in the NOR test [37]. Thus, overexpression of miR-137 in the whole brain may be responsible for deficits in sensory gating, sociability, social recognition, visual learning and memory.

Multiple studies indicate that genes predicted as miR-137 targets are enriched as the risk genes of schizophrenia [9, 10, 12, 13, 38]. Interestingly, the down-regulated genes in the postmortem DLPFC of the patients with schizophrenia with short DOI were enriched among the predicted genes for miR-137 (Fig 3A). Furthermore, predicted-altered pathways in the mPFC obtained from the miR-137 Tg mice were partially overlapped with those seen in the postmortem DLPFC of patients with short DOI (Fig 3B). Thus, down-regulated miR-137 pathways may be associated with the emergence and pathophysiology of schizophrenia. On the other hands, transcriptomes of patients with schizophrenia with intermediate and long DOI showed the less overlap with those of miR-137 Tg mice. Schizophrenia is a disease that the relative predominance of symptom presentation changes disease progression [39–41], and antipsychotic drugs are known to alter the expression of many diverse genes [42], thus, the transcriptomes of patients with schizophrenia who had a long-term illness may not have the less overlap with those of miR-137 Tg mice.

Since the human DLPFC and the rodent mPFC are involved in executive functions and working memory [43], transcriptional changes in the mPFC of the miR-137 Tg mice would cause deficits of cognitive function. Interestingly, we found that synaptogenesis signaling pathway, inflammatory-related signaling and inter-cellular-related signaling were highly ranked in the commonly changed pathways between the mPFC in the miR-137 Tg mice and those of the postmortem DLPFC in patients with schizophrenia with short DOI (Table 1). Abnormalities in these three pathways were reported to be involved in deficits of cognitive function in schizophrenia [44–46]. It would be worth testing to explore which pathway is implicated in the deficits of cognitive function in the miR-137 Tg mice.

As mentioned above, we confirmed the psychiatric disorders-associated behavioral deficits and the overlap of predicted-altered pathways between patients with schizophrenia with short DOI and miR-137 Tg mice. Although miR-137 was slightly expressed in the cerebella of the WT mice, the cerebella of the miR-137 Tg mice showed overexpression of miR-137 (Fig 1B). This is because the Thy-1 promoter works in the neuronal cells in the whole brain [22]. In humans, miR-137 is expressed in the whole brain including in the mPFC, striatum, thalamus, and hippocampus, but it is not expressed in the cerebellum [11]. We need to examine the effects of overexpression of miR-137 in the cerebellum later.

Here, we demonstrated that the overexpression of miR-137 in the whole brain induces the several phenotypes that are relevant to aspects of psychiatric disorders, such as schizophrenia at the behavioral and molecular levels. Although the increased miR-137 in our model is opposite direction to the observed effect of the risk allele in the study with human postmortem brain [11], our results provide new insight into the mechanistic understanding of miR-137 function in the emergence and pathophysiology of schizophrenia. Furthermore, the miR-137 Tg mice appear to be a useful tool for researching the pathophysiology of psychiatric disorders.

## Supporting information

S1 Table Transcriptional changes in several brain regions in miR-137 Tg mice. (XLSX)

S2 Table Mmu-miR-137 putative target genes selected by miRWalk ver. 2.0. (XLSX)

S3 Table Genes overlapping between transcriptional changes in miR-137 Tg mice and mmu-miR-137 putative target genes. (XLSX)

S4 Table Transcriptional changes in patients with schizophrenia with different DOI. (XLSX)

S5 Table Hsa-miR-137 putative target genes selected by miRWalk ver. 2.0. (XLSX)

S6 Table Genes overlapping between transcriptional changes in patients with schizophrenia and hsa-miR-137 putative target genes. (XLSX)

S7 Table Common pathways between miR-137 Tg mice and patients who died in equal to or less than 4 years after initial diagnosis of schizophrenia. (XLSX)

S1 Fig Relative expression of miR-137 in Tg mice. Expression level of miR-137 in the several brain regions in WT and miR-137 Tg mice. mPFC, medial PFC; Str, striatum; Thal, thalamus; Hip, hippocampus; Cb, cerebellum. Data are expressed as the mean + SEM (10-month-old male mice, n = 3).
(PDF)

## References

[1] Freedman R. Schizophrenia. N Engl J Med. 2003; 349(18):1738–49. doi: 10.1056/NEJMra035458 14585943

[2] International Schizophrenia Consortium, Purcell SM, Wray NR, Stone JL, Visscher PM, O'Donovan MC, et al. Common polygenic variation contributes to risk of schizophrenia and bipolar disorder. Nature. 2009; 460(7256):748–52. doi: 10.1038/nature08185 19571811 PMC3912837

[3] Davis J, Eyre H, Jacka FN, Dodd S, Dean O, McEwen S, et al. A review of vulnerability and risks for schizophrenia: Beyond the two hit hypothesis. Neurosci Biobehav Rev. 2016; 65:185–94. doi: 10.1016/j.neubiorev.2016.03.017 27073049 PMC4876729

[4] Arslan A. Imaging genetics of schizophrenia in the post-GWAS era. Prog Neuropsychopharmacol Biol Psychiatry. 2018; 80(Pt B):155–65. doi: 10.1016/j.pnpbp.2017.06.018 28645536

[5] Dong D, Wang Y, Chang X, Luo C, Yao D. Dysfunction of Large-Scale Brain Networks in Schizophrenia: A Meta-analysis of Resting-State Functional Connectivity. Schizophr Bull. 2018; 44(1):168–81. doi: 10.1093/schbul/sbx034 28338943 PMC5767956

[6] Lošák J, Hüttlová J, Lipová P, Marecek R, Bareš M, Filip P, et al. Predictive Motor Timing and the Cerebellar Vermis in Schizophrenia: An fMRI Study. Schizophr Bull. 2016; 42(6):1517–27. doi: 10.1093/schbul/sbw065 27190280 PMC5049535

[7] International Schizophrenia Consortium. Rare chromosomal deletions and duplications increase risk of schizophrenia. Nature. 2008; 455(7210):237–41. doi: 10.1038/nature07239 18668038 PMC3912847

[8] International Schizophrenia Consortium, Purcell SM, Wray NR, Stone JL, Visscher PM, O'Donovan MC, et al. Common polygenic variation contributes to risk of schizophrenia and bipolar disorder. Nature. 2009; 460(7256):748–52. doi: 10.1038/nature08185 19571811 PMC3912837

[9] Schizophrenia Psychiatric Genome-Wide Association Study (GWAS) Consortium. Genome-wide association study identifies five new schizophrenia loci. Nat Genet. 2011; 43(10):969–76. doi: 10.1038/ng.940 21926974 PMC3303194

[10] Schizophrenia Working Group of the Psychiatric Genomic Consortium. Biological insights from 108 schizophrenia-associated genetic loci. Nature. 2014; 511(7510):421–27. doi: 10.1038/nature13595 25056061 PMC4112379

[11] Guella I, Sequeira A, Rollins B, Morgan L, Torri F, van Erp TG, et al. Analysis of miR-137 expression and rs1625579 in dorsolateral prefrontal cortex. J Psychiatr Res. 2013; 47(9):1215–21. doi: 10.1016/j.jpsychires.2013.05.021 23786914 PMC3753093

[12] Kwon E, Wang W, Tsai LH. Validation of schizophrenia-associated genes CSMD1, C10orf26, CACNA1C and TCF4 as miR-137 targets. Mol Psychiatry. 2013; 18(1):11–2. doi: 10.1038/mp.2011.170 22182936

[13] Mahmoudi E, Cairns MJ. MiR-137: an important player in neural development and neoplastic transformation. Mol Psychiatry. 2017; 22(1):44–55. doi: 10.1038/mp.2016.150 27620842 PMC5414082

[14] Warburton A, Breen G, Bubb VJ, Quinn JP. A GWAS SNP for schizophrenia is linked to the internal miR137 promoter and supports differential allele-specific expression. Schizophr Bull. 2016; 42(4):1003–8. doi: 10.1093/schbul/sbv144 26429811 PMC4903043

[15] Forrest MP, Zhang H, Moy W, Mc Gowan H, Leites C, Dionisio LE, et al. Open chromatin profiling in hiPSC-derived neurons prioritizes functional noncoding psychiatric risk variants and highlights neurodevelopmental loci. Cell Stem Cell. 2017; 21 (3):305–18. doi: 10.1016/j.stem.2017.07.008 28803920 PMC5591074

[16] Green MJ, Cairns MJ, Wu J, Dragovic M, Jablensky A, Tooney PA, et al. Genome-wide supported variant MIR137 and severe negative symptoms predict membership of an impaired cognitive subtype of schizophrenia. Mol Psychiatry. 2013; 18(7):774–80. doi: 10.1038/mp.2012.84 22733126

[17] Lett TA, Chakavarty MM, Felsky D, Brandl EJ, Tiwari AK, Gonçalves VF, et al. The genome-wide supported microRNA-137 variant predicts phenotypic heterogeneity within schizophrenia. Mol Psychiatry. 2013; 18(4):443–50. doi: 10.1038/mp.2013.17 23459466

[18] Siegert S, Seo J, Kwon EJ, Rudenko A, Cho S, Wang W, et al. The schizophrenia risk gene product miR-137 alters presynaptic plasticity. Nat Neurosci. 2015; 18(7):1008–16. doi: 10.1038/nn.4023 26005852 PMC4506960

[19] Santarelli DM, Beveridge NJ, Tooney PA, Cairns MJ. Upregulation of dicer and microRNA expression in the dorsolateral prefrontal cortex Brodmann area 46 in schizophrenia. Biol Psychiatry. 2011; 69:180–87. doi: 10.1016/j.biopsych.2010.09.030 21111402

[20] Wu S, Zhang R, Nie F, Wang X, Jiang C, Liu M, et al. MicroRNA-137 inhibits EFNB2 expression affected by a genetic variant and is expressed aberrantly in peripheral blood of schizophrenia patients. EBioMedicine. 2016; 12:133–42. doi: 10.1016/j.ebiom.2016.09.012 27650867 PMC5078603

[21] Liu S, Zhang F, Wang X, Shugart YY, Zhao Y, Li X, et al. Diagnostic value of blood-derived microRNAs for schizophrenia: results of a meta-analysis and validation. Sci Rep. 2017; 7(1):15328. doi: 10.1038/s41598-017-15751-5 29127368 PMC5681644

[22] Vidal M, Morris R, Grosveld F, Spanopoulou E. Tissue-specific control elements of the Thy-1 gene. EMBO J. 1990; 9(3):833–40. 1968831 10.1002/j.1460-2075.1990.tb08180.x PMC551743

[23] Caroni P. Overexpression of growth-associated proteins in the neurons of adult transgenic mice. J Neurosci Methods. 1997; 71(1):3–9. 9125370 10.1016/s0165-0270(96)00121-5

[24] Bartel DP. MicroRNAs: target recognition and regulatory functions. Cell. 2009; 136(2):215–33. doi: 10.1016/j.cell.2009.01.002 19167326 PMC3794896

[25] Dweep H, Sticht C, Pandey P, Gretz N. miRWalk—database: prediction of possible miRNA binding sites by "walking" the genes of three genomes. J Biomed Inform. 2011; 44(5):839–47. doi: 10.1016/j.jbi.2011.05.002 21605702

[26] Kupershmidt I, Su QJ, Grewal A, Sundaresh S, Halperin I, Flynn J, et al. Ontology-based meta-analysis of global collections of high-throughput public data. PLoS One. 2010; 5(9). pii: e13066. 10.1371/journal.pone.0013066 PMC2947508 20927376

[27] Powell SB, Weber M, Geyer MA. Genetic models of sensorimotor gating: relevance to neuropsychiatric disorders. Curr Top Behav Neurosci. 2012; 12:251–318. doi: 10.1007/7854_2011_195 22367921 PMC3357439

[28] Wilson CA, Koenig JI. Social interaction and social withdrawal in rodents as readouts for investigating the negative symptoms of schizophrenia. Eur Neuropsychopharmacol. 2014; 24(5):759–73. doi: 10.1016/j.euroneuro.2013.11.008 24342774 PMC4481734

[29] Yang SS, Huang CL, Chen HE, Tung CS, Shih HP, Liu YP. Effects of SPAK knockout on sensorimotor gating, novelty exploration, and brain area-dependent expressions of NKCC1 and KCC2 in a mouse model of schizophrenia. Prog Neuropsychopharmacol Biol Psychiatry. 2015; 61:30–6. doi: 10.1016/j.pnpbp.2015.03.007 25797415

[30] Weinberger DR, Berman KF, Zec RF. Physiologic dysfunction of dorsolateral prefrontal cortex in schizophrenia. I. Regional cerebral blood flow evidence. Arch Gen Psychiatry. 1986; 43(2):114–24. doi: 10.1001/archpsyc.1986.01800020020004 3947207

[31] Brown VJ, Bowman EM. Rodent models of prefrontal cortical function. Trends Neurosci. 2002; 25(7):340–3. 12079756 10.1016/s0166-2236(02)02164-1

[32] Narayan S, Tang B, Head SR, Gilmartin TJ, Sutcliffe JG, Dean B, et al. Molecular profiles of schizophrenia in the CNS at different stages of illness. Brain Res. 2008; 1239:235–48. doi: 10.1016/j.brainres.2008.08.023 18778695 PMC2783475

[33] Swerdlow NR, Light GA. Animal models of deficient sensorimotor gating in schizophrenia: Are they still relevant? Curr Top Behav Neurosci. 2016; 28:305–25. doi: 10.1007/7854_2015_5012 27311762

[34] Yizhar O, Fenno LE, Prigge M, Schneider F, Davidson TJ, O’Shea DJ, et al. Neocortical excitation/inhibition balance in information processing and social dysfunction. Nature. 2011; 477(7363):171–8. doi: 10.1038/nature10360 21796121 PMC4155501

[35] Kennedy DP, Adolphs R. The social brain in psychiatric and neurological disorders. Trends Cogn Sci. 2012; 16(11):559–72. doi: 10.1016/j.tics.2012.09.006 23047070 PMC3606817

[36] Young JW, Powell SB, Risbrough V, Marston HM, Geyer MA. Using the MATRICS to guide development of a preclinical cognitive test battery for research in schizophrenia. Pharmacol Ther. 2009; 122(2):150–202. doi: 10.1016/j.pharmthera.2009.02.004 19269307 PMC2688712

[37] Tanimizu T, Kono K, Kida S. Brain networks activated to form object recognition memory. Brain Res Bull. 2018; 141:27–34. doi: 10.1016/j.brainresbull.2017.05.017 28587862

[38] Wright C, Calhoun VD, Ehrlich S, Wang L, Tumer JA, Bizzozero NI. Meta gene set enrichment analyses link miR-137-regulated pathways with schizophrenia risk. Front Genet. 2015; 6:147. doi: 10.3389/fgene.2015.00147 25941532 PMC4403556

[39] Liberman JA, Perkins D, Belger A, Chakos M, Jarkog F, Boteva K et al. The early stages of schizophrenia: speculations on pathogenesis, pathophysiology, and therapeutic approaches. Biol Psychiatry 2001; 50(11):884–897. 11743943 10.1016/s0006-3223(01)01303-8

[40] McGlashan TH. The profiles of clinical deterioration in schizophrenia. J Psychiatr Res 1998; 32(3–4):133–41. 9793866 10.1016/s0022-3956(97)00015-0

[41] Kurtz MM. Neurocognitive impairment across the lifespan in schizophrenia: an update. Schizophr Res. 2005; 74(1):15–26. doi: 10.1016/j.schres.2004.07.005 15694750

[42] Thomas EA. Molecular profiling of antipsychotic drug function: convergent mechanisms in the pathology and treatment of psychiatric disorders. Mol Neurobiol 2006; 34(2): 109–28. doi: 10.1385/MN:34:2:109 17220533

[43] Mervis JE., Capizzi RJ, Boroda E, MacDonald AW 3rd. Transcranial direct current stimulation over the dorsolateral prefrontal cortex in schizophrenia: a quantitative review of cognitive outcomes. Front Hum Neurosci. 2017; 11:44. doi: 10.3389/fnhum.2017.00044 28210217 PMC5288642

[44] Ahmed AO, Bhat IA. Psychopharmacological treatment of neurocognitive deficits in people with schizophrenia: a review of old and new targets. CNS Drugs. 2014; 28(4):301–18. doi: 10.1007/s40263-014-0146-6 24526625

[45] Khandaker GM, Cousins L, Deakin J, Lennox BR, Yolken R, Jones PB. Inflammation and immunity in schizophrenia: implications for pathophysiology and treatment. Lancet Psychiatry. 2015; 2(3):258–70. doi: 10.1016/S2215-0366(14)00122-9 26359903 PMC4595998

[46] Krystal JH, Anticevic A, Yang GJ, Dragoi G, Driesen NR, Wang XJ, et al. Impaired tuning of neural ensembles and the pathophysiology of schizophrenia: a translational and computational neuroscience perspective. Biol Psychiatry. 2017; 81(10):874–85. doi: 10.1016/j.biopsych.2017.01.004 28434616 PMC5407407

