# Supplementary figures and images for: Transgenic mice overexpressing miR-137 in the brain show schizophrenia-associated behavioral deficits and transcriptome profiles
Source: PLoS One. 2019 Jul 30;14(7):e0220389. doi: 10.1371/journal.pone.0220389 (PMC6667145; doi:10.1371/journal.pone.0220389)

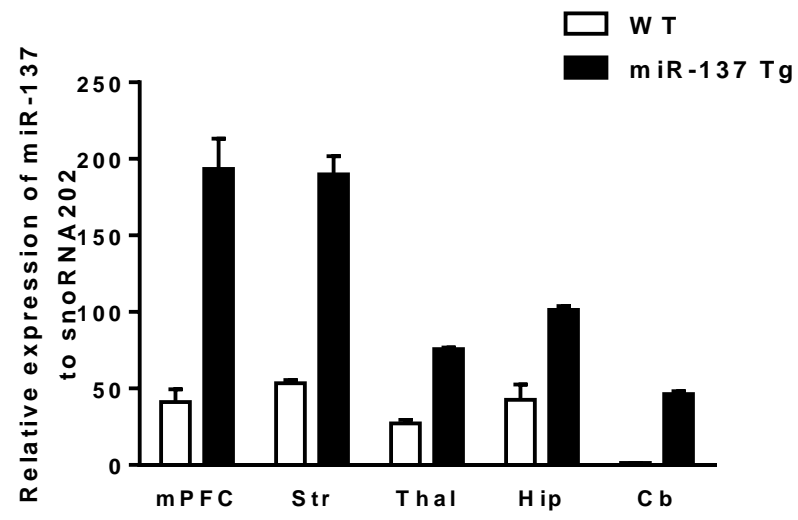

Supplement: S1 Fig — Expression level of miR-137 in the several brain regions in WT and miR-137 Tg mice. mPFC, medial PFC; Str, striatum; Thal, thalamus; Hip, hippocampus; Cb, cerebellum. Data are expressed as the mean + SEM (10-month-old male mice, n = 3). (PDF) [file pone.0220389.s008.pdf]
